# Correction: Impact of patients’ age and comorbidities on prostate cancer overdiagnosis in clinical practice

**DOI:** 10.1371/journal.pone.0339626

**Published:** 2025-12-23

**Authors:** Abraham Beltrán, Lucy Parker, Irene Moral Peláez, Juan Pablo Caballero-Romeu, Elisa Chilet-Rosell, Ildefonso Hernández-Aguado, Pablo Alonso-Coello, Elena Ronda, Luis Gómez-Pérez, Blanca Lumbreras

The third author’s name is spelled incorrectly. The correct name is: Irene Moral Peláez. The correct citation is: Beltrán A, Parker L, Moral Peláez I, Caballero-Romeu JP, Chilet-Rosell E, Hernández-Aguado I, et al. (2025) Impact of patients’ age and comorbidities on prostate cancer overdiagnosis in clinical practice. PLoS ONE 20(2): e0315979. https://doi.org/10.1371/journal.pone.0315979.
